# DUX4 induces a transcriptome more characteristic of a less-differentiated cell state and inhibits myogenesis

**DOI:** 10.1242/jcs.180372

**Published:** 2016-10-15

**Authors:** Paul Knopp, Yvonne D. Krom, Christopher R. S. Banerji, Maryna Panamarova, Louise A. Moyle, Bianca den Hamer, Silvère M. van der Maarel, Peter S. Zammit

**Affiliations:** 1 Randall Division of Cell and Molecular Biophysics, Faculty of Life Sciences and Medicine, New Hunt's House, King's College London, Guy's Campus, London SE1 1UL, UK; 2 Department of Human Genetics, Leiden University Medical Center, Leiden, Postbus 9600, 2300 RC, The Netherlands; 3 Centre of Mathematics and Physics in the Life Sciences and Experimental Biology, University College London, London WC1E 6BT, UK

**Keywords:** DUX4, DUX4c, FSHD, Stem cells, Skeletal muscle, Satellite cell, Transcriptome

## Abstract

Skeletal muscle wasting in facioscapulohumeral muscular dystrophy (FSHD) results in substantial morbidity. On a disease-permissive chromosome 4qA haplotype, genomic and/or epigenetic changes at the D4Z4 macrosatellite repeat allows transcription of the *DUX4* retrogene. Analysing transgenic mice carrying a human D4Z4 genomic locus from an FSHD-affected individual showed that DUX4 was transiently induced in myoblasts during skeletal muscle regeneration. Centromeric to the D4Z4 repeats is an inverted D4Z4 unit encoding DUX4c. Expression of DUX4, DUX4c and DUX4 constructs, including constitutively active, dominant-negative and truncated versions, revealed that DUX4 activates target genes to inhibit proliferation and differentiation of satellite cells, but that it also downregulates target genes to suppress myogenic differentiation. These transcriptional changes elicited by DUX4 in mouse have significant overlap with genes regulated by DUX4 in man. Comparison of DUX4 and DUX4c transcriptional perturbations revealed that DUX4 regulates genes involved in cell proliferation, whereas DUX4c regulates genes engaged in angiogenesis and muscle development, with both DUX4 and DUX4c modifing genes involved in urogenital development. Transcriptomic analysis showed that DUX4 operates through both target gene activation and repression to orchestrate a transcriptome characteristic of a less-differentiated cell state.

## Abstract (precis)

**Summary:** DUX4 underlies pathogenesis in facioscapulohumeral muscular dystrophy. DUX4 acts mainly as a transcriptional activator that inhibits myogenesis by orchestrating a gene expression profile representative of a more stem-cell-like state.

## INTRODUCTION

Facioscapulohumeral muscular dystrophy (FSHD) is autosomal dominant, characterised by descending, often asymmetric, skeletal muscle weakness and wasting, starting with facial, shoulder and proximal upper limb muscles (Tawil and Van Der Maarel, 2006). FSHD has an incidence of ∼1:15,000 (Flanigan et al., 2001) and prevalence of 1 in 8333 to 1 in 20,000 (Deenen et al., 2014; Padberg et al., 1995).

Satellite cells are responsible for maintenance and repair of skeletal muscle (Relaix and Zammit, 2012), and muscle dystrophy implies a failure of this normal homeostatic and repair function (Morgan and Zammit, 2010). Consistent with this premise, myoblasts from FSHD-affected individuals are more susceptible to oxidative stress and show deregulation of *MYOD* (also known as *MYOD1*) (Winokur et al., 2003a,b), and differentiate into myotubes with abnormal morphology (Barro et al., 2008).

In 95% of FSHD cases (FSHD1; OMIM158900), a contraction to 1–10 units and CpG-DNA hypomethylation of the highly polymorphic D4Z4 repeat region in the subtelomere of chromosome 4q occurs (van Deutekom et al., 1993; van Overveld et al., 2003; Wijmenga et al., 1992). Each D4Z4 repeat contains an open reading frame (ORF) for Double homeobox 4 (*DUX4*) (OMIM606009) (Gabriels et al., 1999; Hewitt et al., 1994), and DNA-CpG hypomethylation is associated with *DUX4* transcription from the D4Z4 units, which are usually somatically repressed (Dixit et al., 2007). A polymorphism in disease-permissive 4qA haplotypes provides a polyadenylation signal for *DUX4* transcripts emanating from the final D4Z4 unit (Lemmers et al., 2010). The remaining 5% (FSHD2; OMIM158901) have no contraction of the D4Z4 repeats but still exhibit CpG-DNA hypomethylation of D4Z4 units and also carry a permissive 4qA allele. Most FSHD2 individuals have mutations in the chromatin-modifying protein SMCHD1 (Lemmers et al., 2012), whereas others have mutations in the DNA methyltransferase DNMT3B (van den Boogaard et al., 2016). Although altered expression of non-coding RNAs (Cabianca et al., 2012) and neighbouring 4q genes – e.g. *FRG1* (Gabellini et al., 2006) and mutations in *FAT1* (Caruso et al., 2013) – have also been implicated in FSHD, there is growing consensus that aberrant expression of DUX4 underlies pathogenesis in both FSHD1 and FSHD2, acting with a gain-of-function mechanism (Tawil et al., 2014).

DUX4 mRNA and/or protein can be detected in FSHD-individual-derived proliferating myoblasts, with levels increasing during differentiation and sporadic expression in rare nuclei of myotubes (Dixit et al., 2007; Jones et al., 2012; Kowaljow et al., 2007; Snider et al., 2010; Tassin et al., 2013). A DUX4 reporter reveals that DUX4 is transcriptionally active in FSHD-derived proliferating myoblasts, which becomes more widespread upon myogenic differentiation (Rickard et al., 2015).

D4Z4 tandem repeats and *DUX4* ORF are evolutionarily conserved in placental mammals (Clapp et al., 2007; Giussani et al., 2012). Identification of DUX proteins in germline cells (Geng et al., 2012) suggests a role during development, but little is known of endogenous DUX4 function. Two important DUX4 isoforms are derived from the D4Z4 ORF – DUX4-fl (full-length) that is expressed in germline and stem cells, and the alternatively spliced DUX4-s (short) isoform expressed in some somatic cells at low levels (Snider et al., 2010).

Mice transgenic for a D4Z4 repeat array from an FSHD individual recapitulate epigenetic phenomena consistent with a contracted FSHD locus. *DUX4* is expressed in germline cells, and the protein can be detected in myoblasts and muscle, but there is no overt skeletal muscle pathology (Krom et al., 2013). Ectopic DUX4 expression results in impaired myogenesis (Dandapat et al., 2014) and gross muscle damage through p53-dependent apoptosis in other mouse models (Wallace et al., 2010).

How incomplete repression of DUX4 in somatic cells causes muscular dystrophy is enigmatic. DUX4 inhibits muscle differentiation and induces myoblast death (Bosnakovski et al., 2008a; Kowaljow et al., 2007). DUX4 also causes myoblasts to differentiate to produce myotubes with a morphology similar to the dysmorphic myotubes from FSHD individuals (Vanderplanck et al., 2011). However, systematic comparison is lacking between DUX4, DUX4c and DUX4-s.

DUX4 is a transcription factor. The N-terminus contains two homeodomains with similarity to those of PAX3 and PAX7 (Bosnakovski et al., 2008b), and the C-terminus is a transcriptional activator (Kawamura-Saito et al., 2006). FSHD muscle biopsies and *DUX4*-expressing myoblast cultures indicate perturbation of Wnt–β-catenin signalling, *MYOD* regulation, oxidative stress and innate immune response (Banerji et al., 2015a; Block et al., 2013; Bosnakovski et al., 2008a; Celegato et al., 2006; Fitzsimons, 2011; Geng et al., 2012; Winokur et al., 2003b). Transcriptome analysis of endogenous DUX4-expressing cells reveals that DUX4 disrupts pathways involved in RNA metabolism, cell signalling, polarity and migration (Rickard et al., 2015), and nonsense-mediated decay (Feng et al., 2015).

Mutation of a DUX4 homeodomain or competitive inhibition by shortened DUX4 splice variants inhibits DUX4 target gene activation and abrogates DUX4-induced cell death (Ferri et al., 2015; Geng et al., 2012; Mitsuhashi et al., 2013; Wallace et al., 2010). Although DUX4 binding motifs have been identified (Dixit et al., 2007; Ferri et al., 2015; Geng et al., 2012; Young et al., 2013; Choi et al., 2016), and ChIP-Seq performed (Geng et al., 2012; Choi et al., 2016), a set of target genes that explains both anti-myogenic and apoptotic phenotypes induced by DUX4 has not been comprehensibly defined.

An incomplete and reversed D4Z4 unit is located 40 kb centromeric to the D4Z4 repeat array. This encodes DUX4c, which lacks the N-terminus and diverges from DUX4-fl in the C-terminal region but is otherwise homologous to DUX4-fl. DUX4c is detectable in FSHD muscle biopsies and FSHD-derived proliferating myoblasts, and increases in myotubes (Ansseau et al., 2009).

Here, we show that DUX4 is transiently elevated in myoblasts during muscle regeneration. To model FSHD, we used retroviral-mediated delivery of *DUX4*, in parallel with truncated, constitutively active and dominant-negative DUX4 versions, as well as with DUX4c. DUX4 activates transcriptional targets to suppress proliferation in satellite cells but can both activate and inhibit transcriptional targets to prevent myogenic differentiation. Transcriptomic analysis showed that DUX4 acts as a strong transcriptional activator but can also inhibit transcriptional targets. *DUX4c* increases transcription of some genes that are induced by *DUX4* but also repressed a significant proportion. In general, DUX4 orchestrates a transcriptome more characteristic of a less-differentiated cell state.

## RESULTS

### DUX4 is transiently expressed during skeletal muscle regeneration

Two transgenic mouse models for FSHD have been previously generated – control D4Z4-12.5 mice contain a human genomic region encompassing 12.5 D4Z4 units, whereas FSHD1 D4Z4-2.5 mice are transgenic for a contracted human repeat with 2.5 D4Z4 units obtained from an FSHD-affected individual. D4Z4-2.5 transgenic mice reveal low and variable levels of DUX4 in skeletal muscles (Krom et al., 2013).

**Fig. 1.**
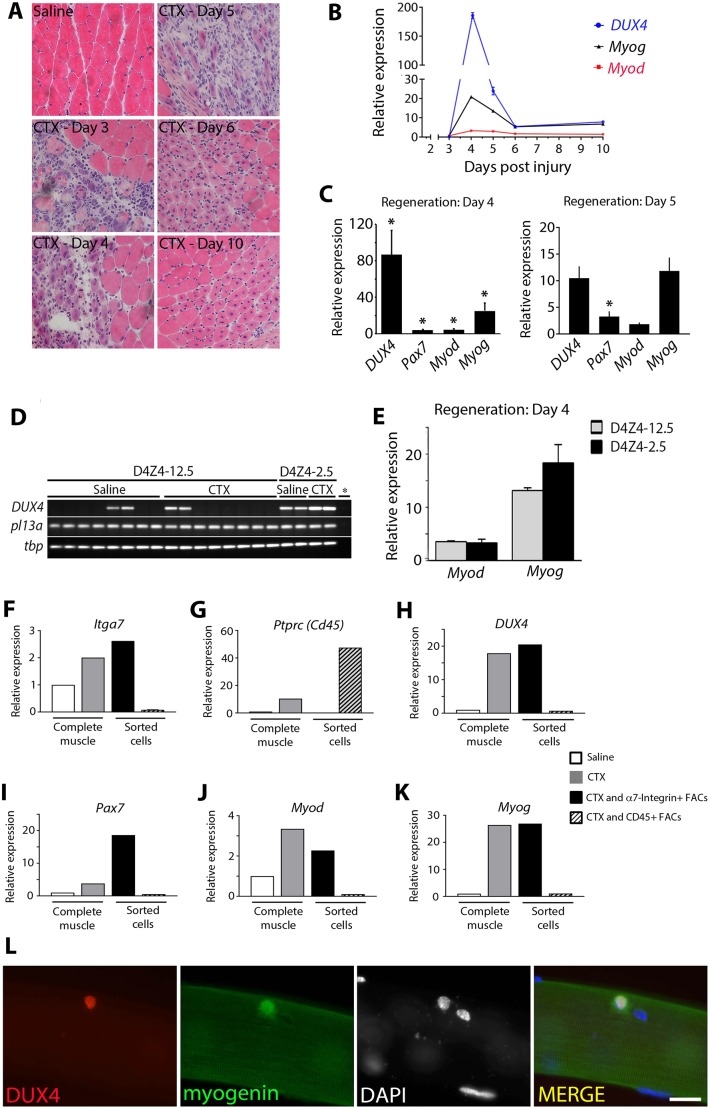
**DUX4 is elevated in transgenic D4Z4-2.5 myoblasts during muscle regeneration.** (A) Gastrocnemius muscles of D4Z4-2.5 mice were injected with cardiotoxin (CTX), whereas the contralateral muscle received saline, and analysed with H&E at 3, 4, 5, 6 and 10 days post-injury. (B) RT-qPCR analysis of human *DUX4* transcribed from the transgenic locus*,* together with *Myod* and *Myog*, was assessed in D4Z4-2.5 mice at 3, 4, 5, 6 and 10 days post-injury. (C) Human *DUX4*, murine *Myod* and *Myog* were assessed with RT-qPCR analysis in gastrocnemius muscle of D4Z4-2.5 mice at day 4 (*n*=3) and day 5 (*n*=3) of regeneration. (D,E) Human *DUX4*, murine *Myod* and *Myog* expression was assessed in gastrocnemius muscle of control D4Z4-12.5 mice, 4 days post injury (*n*=4). (D) *DUX4* in control mice could not be quantified, and so DUX4 products are displayed on a gel (in duplicate), together with reference genes. (E) Quantification of *Myod* and *Myog* expression in regenerating D4Z4-12.5 compared to D4Z4-2.5 muscle. Expression was normalized to that in saline-injected muscle. Data are mean±s.e.m., where an asterisk in C denotes significant difference (*P*<0.05) from saline-injected control using a Student's *t*-test. (F–K) Regenerating D4Z4-2.5 gastrocnemius muscle was isolated 4 days after CTX injection from 14 D4Z4-2.5 mice. 1.5×10^6^ cells were analysed with FACS to isolate the CD31^−^ CD45^−^ SCA1^−^ α7-integrin^+^ population; 5.3×10^6^ cells were analysed for the CD45^+^ population. Saline: RNA from saline-injected D4Z4-2.5 gastrocnemius muscles. RNA from complete saline-injected D4Z4-2.5 gastrocnemius muscle was a negative control, whereas RNA from CTX-injected D4Z4-2.5 gastrocnemius muscle acted as a positive control. (F,G) FACS sorting to enrich for CD45^−^ CD31^−^ SCA1^−^ α7-integrin^+^ cells or CD45^+^ cells was confirmed by performing RT-qPCR. (H) *DUX4* was largely confined to CD45^−^ CD31^−^ SCA1^−^ α7-integrin^+^ cells, identified as myoblasts by (I) *Pax7*, (J) *Myod* and (K) *Myog* expression. Expression values for genes of interest were normalised to those of the reference genes *Tbp* and *Rpl13a*. Expression was normalized to saline-injected muscles. (L) The λ42/L42 construct, used to generate D4Z4-2.5 mice, was transfected into murine satellite cells that were cultured in association with myofibres. At 48 h post transfection, co-immunostaining revealed rare satellite cells containing DUX4 and myogenin protein. Scale bar: 20 µm.

We first screened *DUX4* expression from the human transgenic locus in D4Z4-12.5 (control) and D4Z4-2.5 (FSHD1) mice during skeletal muscle regeneration *in vivo*. Gastrocnemius muscles of five adult D4Z4-12.5 and D4Z4-2.5 mice were injected with cardiotoxin to induce muscle damage, with the contralateral side receiving saline. At days 3, 4, 5, 6 and 10 post-injection, muscle regeneration was analysed by histological examination (Fig. 1A). Skeletal muscle in both D4Z4-12.5 and FSHD1 D4Z4-2.5 mice successfully regenerated, consistent with our previous observations (Krom et al., 2013).

Human *DUX4*, murine *Duxbl*, *Myod* and *Myog* (myogenin) expression was measured using real time quantitative PCR (RT-qPCR) on RNA extracted from the other half of the regenerating gastrocnemius muscles (Fig. 1B; Fig. S1). *Myog* levels increased during the early phase of muscle regeneration in both D4Z4-2.5 and D4Z4-12.5 mice as expected. As shown previously (Wu et al., 2014), substantial *Duxbl* levels were detectable in mouse skeletal muscle, with levels enhanced during regeneration (Fig. S1A,B). *DUX4* levels were negligible but increased in gastrocnemius at days 4 and 5 post-cardiotoxin injection of D4Z4-2.5 mice, compared to those in undamaged control muscles, before returning to pre-injury levels at days 6–10 (Fig. 1B)*.* RT*-*qPCR analysis was also performed on RNA from further D4Z4-2.5 gastrocnemius muscles that had regenerated for 4 or 5 days. D4Z4-2.5 muscle at day 4 of regeneration showed a significant increase in *DUX4* levels (*n*=3 mice) and approached significance at day 5 (Fig. 1C), with control genes *Pax7*, *Myod* and *Myog* generally higher than in undamaged muscle, as expected. However, *DUX4* transcripts could not generally be detected in either undamaged or regenerating muscle from control D4Z4-12.5 mice (Fig. 1D,E). Thus, in FSHD1 D4Z4-2.5 mice, DUX4 expression increases transiently during early muscle regeneration *in vivo*.

### DUX4 is expressed in myoblasts during skeletal muscle regeneration

To determine if DUX4 is expressed in myoblasts during muscle regeneration, fluorescence-activated cell sorting (FACS) was performed in a pilot experiment at day 4 post cardiotoxin injection – the time point with the highest levels of *DUX4* transcripts (Fig. 1C). *DUX4* could be detected in RNA pooled from eight regenerating muscles and the FACS-isolated CD31^−^ CD45^−^ SCA1^−^ α7-integrin^+^ population, which was identified as a myoblast population because they also expressed *Pax7*, *Myod* and *Myog* (Fig. S1C).

To confirm that *DUX4* expression was confined to myoblasts and not inflammatory cells, FACS was performed to isolate the CD31^−^ CD45^−^ SCA1^−^ α7-integrin^+^ population or CD45^+^ cells (haematopoetic lineage) from a pool of 14 gastrocnemius muscles from D4Z4-2.5 mice after 4 days of regeneration. Purity was confirmed by quantifying gene expression for α7-integrin (*Itga7*) and CD45 (encoded by *Ptprc*) (Fig. 1F,G). *DUX4* was largely confined to the CD45^−^ CD31^−^ SCA1^−^ α7-integrin^+^ cell population (Fig. 1H), identified as myoblasts through *Pax7*, *Myod* and *Myog* expression, but DUX4 was not present in CD45^+^ cells (Fig. 1H–K).

The λ42/L42 construct (van Deutekom et al., 1993) used to generate the D4Z4-2.5 transgenic mice was also transfected into wild-type murine satellite cells, and rare DUX4-protein-containing satellite cells could be identified (Fig. 1L). Thus, the native human contracted D4Z4 repeat containing 5′ and 3′ regions can be regulated in murine satellite cells to produce DUX4 protein *in vivo* and *in vitro*.

### The mechanism of action of DUX4

*DUX4* that is transcribed from the potential upstream Met-Lys-Gly (MKG) start site, or from the originally identified Met-Ala-Leu (MAL) start site, encodes a protein that inhibits myogenic differentiation and induces cell death (Snider et al., 2009). DUX4c is identical to DUX4 (MAL start) in the N-terminus and across the double homeodomain but has an alternative 32-amino-acid C-terminus. DUX4c and DUX4 proteins lacking the C-terminus inhibit differentiation but do not induce overt cell death (Ansseau et al., 2009; Bosnakovski et al., 2008a). Interestingly, the DUX4 C-terminal peptide alone inhibits muscle differentiation (Snider et al., 2009).

**Fig. 2.**
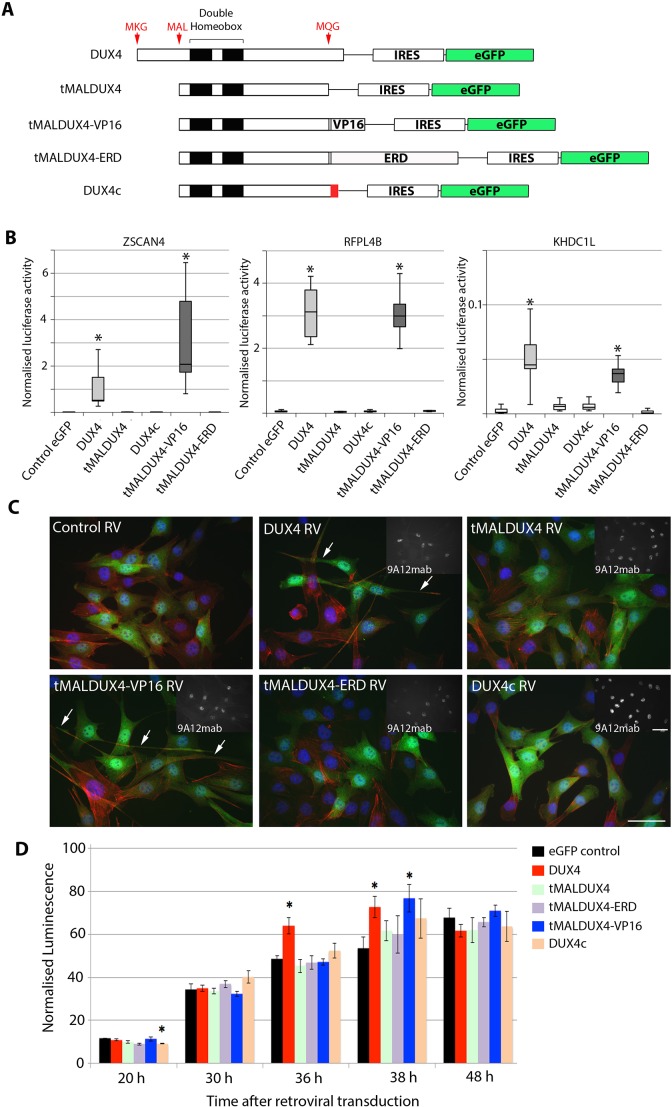
**DUX4 changes cell morphology and increases apoptosis.** (A) Schematic showing the DUX4 constructs and DUX4c, together with IRES-eGFP. (B) Transcriptional activity was assessed in C2C12 myoblasts by co-transfection of DUX4 constructs and DUX4c with three DUX4-responsive promoters driving luciferase reporter genes (pZSCAN4-luc, pKHDC1L-luc or *RFPL4b-luc*), together with β-galactosidase for transfection normalisation. Only DUX4 and tMALDUX4–VP16 strongly activated DUX4 reporters. Boxes represent the interquartile range (central 50% of data) with the median indicated by a line, and whiskers indicate the extremes of the distribution.(C) Retroviral (RV)-mediated expression of DUX4 constructs and DUX4c in C2C12 myoblasts that had been co-immunostained for eGFP (green) to identify transduced cells, actin (red) and DUX4 (white, inset panel) with DAPI (blue). DUX4- and tMALDUX4–VP16-transduced myoblasts had altered morphology, with long projections (arrows). Scale bars: 20 µm. (D) Apoptosis was assayed in plated satellite-cell-derived primary myoblasts by measuring caspase 3 and caspase 7 activity over 48 h post transduction with retroviruses encoding DUX4 constructs and DUX4c. Data are mean±s.e.m. from three experiments (B) or four mice (D), where an asterisk denotes significant difference (*P*<0.05) from GFP control using a Student's *t*-test.

We used retroviral expression vectors encoding DUX4, DUX4c or a truncated DUX4 variant termed tMALDUX4 that initiates at the MAL start site and is intact across the two homeodomains but terminates at the Met-Gln-Gly (MQG) site, so lacks the C-terminal 75 amino acids of DUX4 or the 32 amino acids of DUX4c (Snider et al., 2009). We also used tMALDUX4 fused to a VP16 transactivation domain to generate the constitutively active tMALDUX4–VP16 construct, or the Engrailed repressor domain to create the dominant-negative tMALDUX4–ERD construct (Banerji et al., 2015a) (Fig. 2A).

To assess transcriptional activation of our DUX4 constructs, we used three DUX4 reporter constructs incorporating the *ZSCAN4*, *RFPL4b* or *KHDC1L* promoters driving a luciferase reporter gene (Ferreboeuf et al., 2014). DUX4 constructs and DUX4 reporters were co-transfected into murine C2C12 myoblasts, together with an RSV-β-galactosidase construct for normalisation of transfection efficiency. DUX4 and tMALDUX4–VP16 robustly activated all three DUX4 reporters compared to transfection with control plasmid, whereas tMALDUX4, DUX4c or tMALDUX4–ERD did not (Fig. 2B). tMALDUX4–VP16 activated the *ZSCAN4* reporter more than DUX4, whereas *RFPL4b* and *KHDC1L* reporters were activated to similar extents by both constructs.

### DUX4 alters cell morphology and causes apoptosis through transcriptional activation of target genes

Proteins encoded by each *DUX4* construct could be identified in C2C12 myoblast nuclei using the 9A12 monoclonal antibody (Dixit et al., 2007). The viral vector has an IRES-eGFP module to mark transduced cells (Fig. 2C). C2C12 myoblasts that were transduced with *DUX4* displayed a specific morphological phenotype, extending long cytoplasmic projections (Fig. 2C), as previously observed in the iC2C12-*DUX4* immortalised cell line (Bosnakovski et al., 2008b). Expression of tMALDUX4–VP16 also caused long cytoplasmic projections, but tMALDUX4–ERD, tMALDUX4 or DUX4c did not perturb morphology, indicating that the projections are a result of transcriptional activation of target genes.

We next assayed apoptosis in plated satellite-cell-derived primary myoblasts by measuring caspase 3 and caspase 7 activity over the 48-h period after transduction with retroviruses encoding the DUX4 constructs. Caspase 3 and caspase 7 activity generally increased over time, as expected (Dee et al., 2002). However, further increased caspase activity was measured at 36 and 38 h post transduction in myoblasts expressing DUX4, and in those expressing tMALDUX4–VP16 at 38 h (Fig. 2D).

### DUX4 maintains Pax7 expression through transcriptional activation of target genes

**Fig. 3.**
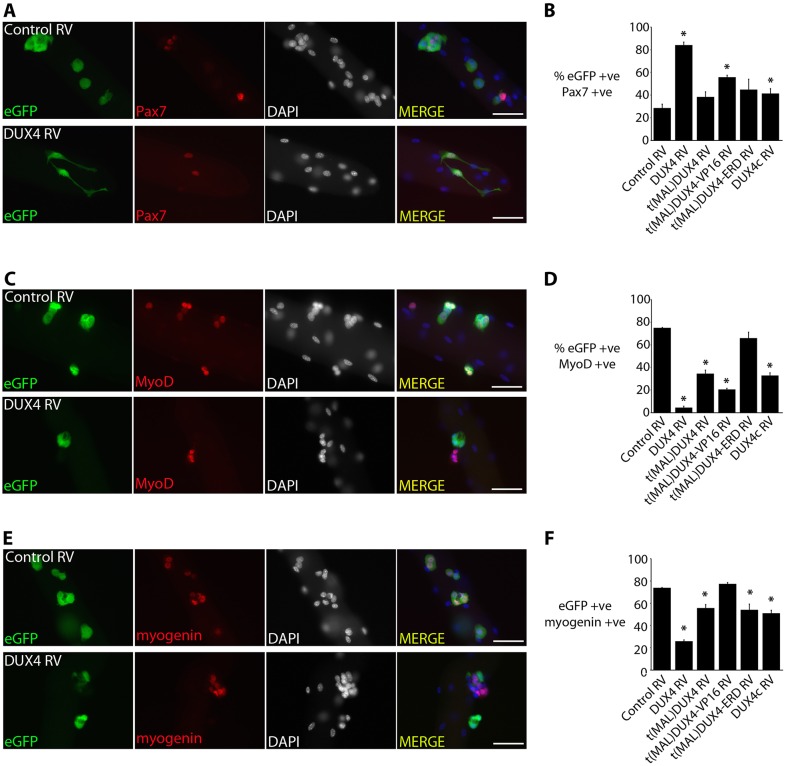
**DUX4 inhibits myogenic progression.** Satellite cells maintained on their associated myofibres were transduced with retroviruses (RVs) encoding DUX4 constructs, DUX4c or control, and were cultured for 48 h and co-immunostained for eGFP and Pax7, eGFP and MyoD or eGFP and myogenin. (A,C,E) Co-immunostaining with control and DUX4-encoding retrovirus only, is illustrated. (B) The proportion increased of DUX4 and tMALDUX4–VP16 (eGFP) satellite cells expressing Pax7. (D) DUX4 and tMALDUX4–VP16 reduced the proportion of cells expressing MyoD. Pax7 (B) and MyoD (D) were unaffected by tMALDUX4–ERD. (F) DUX4 but not tMALDUX4–VP16 reduced the proportion of cells expressing myogenin. Both tMALDUX4 and DUX4c reduced the proportion of cells with MyoD (D) and myogenin (F), but only DUX4c affected Pax7 expression (B). Data are mean±s.e.m. from three mice; an asterisk denotes significant difference (*P*<0.05) from transduction with control RV using a Student's *t*-test. Scale bars: 50 µm.

We first investigated the effects of the DUX4 constructs on early myogenesis. At 24 h after isolation, extensor digitorum longus (EDL) satellite cells that were associated with their myofibres were transduced with either retroviruses encoding DUX4, tMALDUX4, tMALDUX4–VP16, tMALDUX4–ERD, DUX4c or control retrovirus, and were cultured for 48 h before immunostaining (Fig. 3). For illustration, only co-immunostaining for eGFP and Pax7 (Fig. 3A), eGFP and MyoD (Fig. 3C) or eGFP and myogenin (Fig. 3E) after transduction with control or retroviral constructs encoding DUX4 are shown. Similarly, cytoplasmic projections were observed after DUX4 retroviral infection of satellite cells that were associated with EDL myofibres (Fig. 3A).

Quiescent satellite cells express *Pax7*. Upon activation and differentiation of satellite cells, *Pax7* expression decreases, with the few cells retaining Pax7 thought to be those that repopulate the stem cell pool (Zammit et al., 2004). A higher proportion of satellite cells expressing DUX4 and tMALDUX4–VP16 retained Pax7 (Fig. 3A,B). This suggests that DUX4 inhibits myogenic progression in satellite cells and causes retention of proteins that are normally associated with a more naïve stem cell and less-differentiated phenotype.

### DUX4, DUX4c and tMALDUX4 inhibit entry into myogenic differentiation

*Myod* expression increases in proliferating satellite cells and drives the early stages of myogenic differentiation (Zammit et al., 2004). Expression of DUX4 constructs (except tMALDUX4–ERD) significantly reduced the proportion of satellite cells that contained MyoD (Fig. 3C,D).

Satellite cells that have committed to myogenic differentiation express myogenin (Zammit et al., 2004). DUX4 expression significantly reduced the proportion of satellite cells containing myogenin (Fig. 3E,F). DUX4c, tMALDUX4 and tMALDUX4–ERD also reduced the proportion of myogenin-expressing satellite cells, but tMALDUX4–VP16 did not (Fig. 3F).

### The reduction in satellite-cell proliferation is due to DUX4 transcriptional activity

**Fig. 4.**
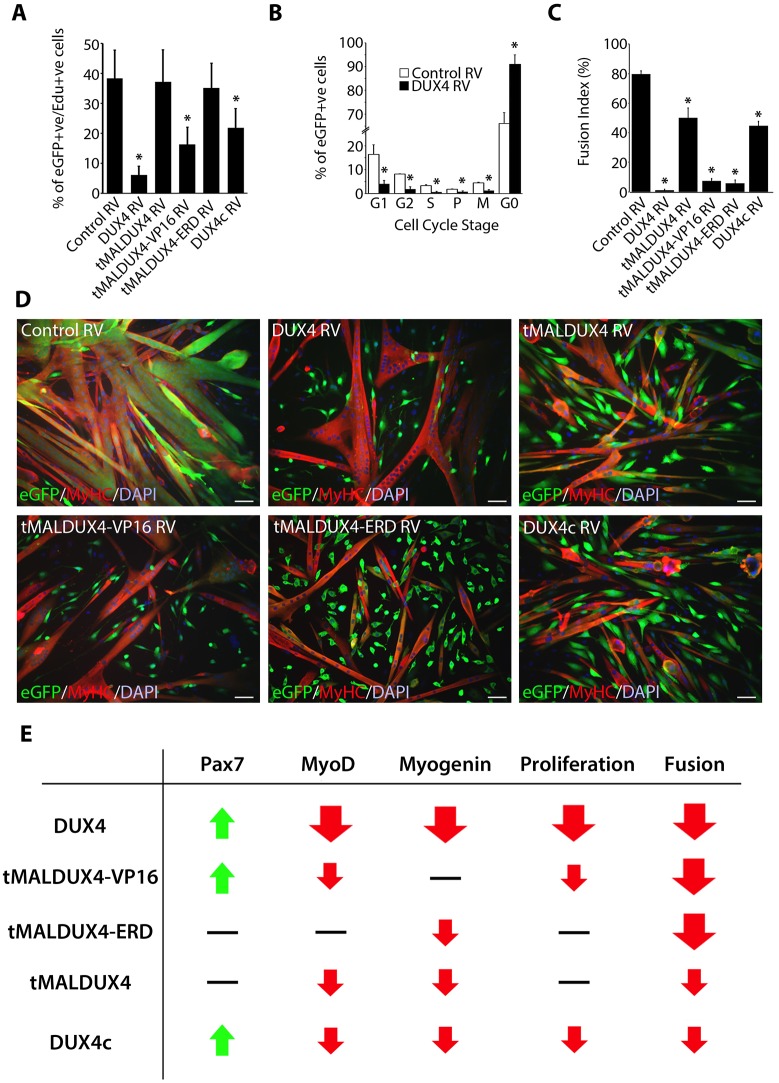
**DUX4 reduces myogenic fusion by both transcriptional activation and suppression of target genes.** Expanded satellite-cell-derived myoblasts were transduced to express DUX4, tMALDUX4, tMALDUX4–VP16, tMALDUX4–ERD, DUX4c or control retrovirus (RV). (A) At 24 h post-transduction, myoblasts were pulsed with EdU for 2 h, fixed and immunostained for eGFP with EdU detection. DUX4, tMALDUX4–VP16 or DUX4c expression reduced the proportion of eGFP+ myoblasts containing EdU. (B) The pattern of phosphorylated histones H1 and H3 immunosignal can be used to identify stages in the cell cycle (Hendzel et al., 1997; Lu et al., 1994) and revealed that DUX4 suppressed cell cycle progression. (C,D) Transduced myoblasts were switched to differentiation medium for 48 h, and co-immunostained for eGFP (green) and MyHC (red) with DAPI counterstain (blue). (C) DUX4 constructs and DUX4c significantly reduced the fusion index (≥2 nuclei). (D) DUX4 constructs reduced the number and size of myotubes, with many unfused eGFP+ and MyHC− myoblasts. Data are mean±s.e.m. from three mice, where an asterisk denotes significant difference (*P*<0.05) from transduction with control RV using a paired Student's *t*-test. Scale bars: 50 µm. (E) Summary of effects of DUX4 constructs and DUX4c on satellite cells.

To examine the effects of DUX4 during proliferation, we used expanded primary myoblast cultures, which were transduced with the *DUX4* retroviral constructs and DUX4c, and pulsed with EdU. There was a reduced proportion of satellite-cell-derived myoblasts containing EdU after transduction with DUX4, tMALDUX4–VP16 or DUX4c constructs, compared to transduction with control retrovirus (Fig. 4A). The proliferation rate was unaltered in myoblasts expressing tMALDUX4 or tMALDUX4–ERD (Fig. 4A). The nuclear pattern of the signal after co-labelling with antibodies against phosphorylated histones H1 and H3 can be used to identify stages of the cell cycle (Hendzel et al., 1997; Lu et al., 1994). DUX4 expression significantly reduced the proportion of satellite cells that were in all phases of the cell cycle and increased the proportion that were in G0 (Fig. 4B).

### Activation or inhibition of DUX4 target genes suppresses myotube formation

We next examined the effects of DUX4 constructs on later phases of differentiation. Satellite-cell-derived myoblasts were cultured at high density to mitigate the anti-proliferative effects of some constructs, transduced with retroviruses encoding DUX4c or DUX4 constructs and switched to low-serum conditions to promote fusion. Co-immunostaining for eGFP and myosin heavy chain (MyHC) revealed that myoblasts that had been infected with control retrovirus readily formed large multinucleated myotubes (fusion index of ≥2 nuclei/myotube), which appear yellow-orange in merged images (Fig. 4C,D). Expression of any of the DUX4 constructs reduced myoblast fusion, resulting in numerous unfused eGFP-positive (green) myoblasts. MyHC-positive but eGFP-negative red myotubes, principally composed of non-transduced myoblasts, could also be identified (Fig. 4D). However, two categories of severity were identified: tMALDUX4 or DUX4c had a less-profound effects on fusion than DUX4, tMALDUX4–VP16 or tMALDUX4–ERD, with cells even unable to differentiate into unfused myocytes expressing MyHC in the latter category (Fig. 4C,D). Thus, both transcriptional activation and suppression of DUX4 target genes reduces and/or prevents myoblast fusion, whereas loss of the C-terminus of DUX4 in tMALDUX4 and DUX4c lessens these inhibitory effects. The effects on satellite cell function of the four DUX4 constructs and DUX4c are summarised in Fig. 4E.

### DUX4 is predominately an activator of transcription

Previous transcriptional profiling of gene expression changes induced by DUX4 constructs (GEO accession number GSE77100; http://www.ncbi.nlm.nih.gov/geo/query/acc.cgi?acc=GSE77100) has revealed that *DUX4* in murine satellite cells recapitulates a transcriptional signature that has been identified in human FSHD muscle biopsies (Banerji et al., 2015a).

**Fig. 5.**
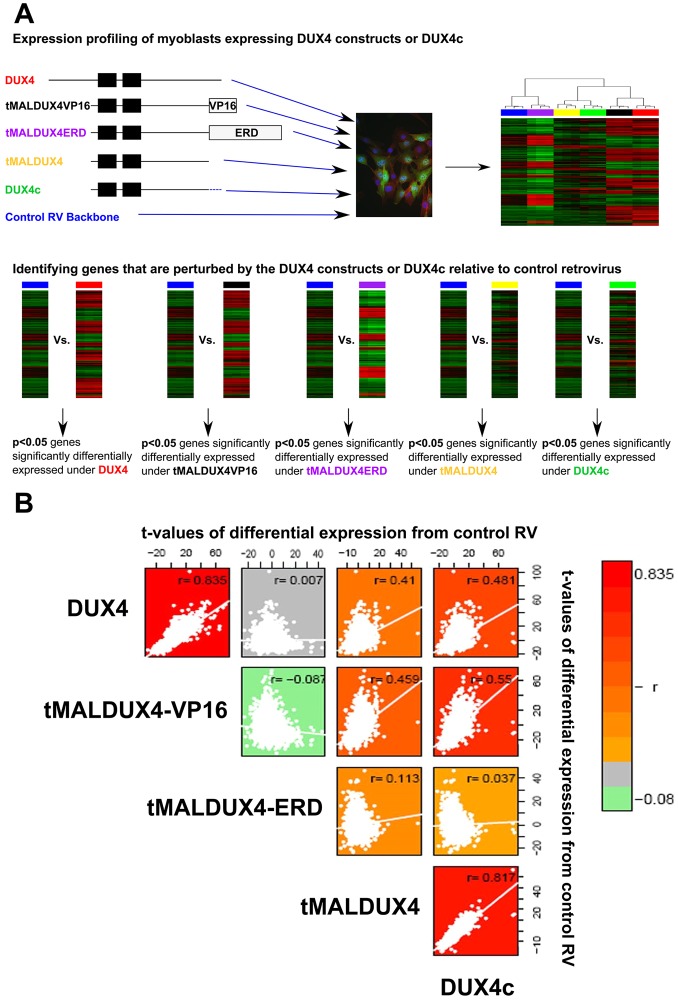
**DUX4 acts by both activating and suppressing target genes.** (A) Flow chart describing the filtering of probes to identify genes whose expression was modified by DUX4 constructs and DUX4c. (B) Global transcriptomic analysis of microarray assays of cells expressing DUX4 constructs (compared to control) demonstrates correlations between differential expression *t*-values. Positive correlations were detected between DUX4 and tMALDUX4–VP16 gene sets and between DUX4 and DUX4c gene sets*.* Lack of anti-correlation between DUX4 and tMALDUX4–ERD gene sets indicates that DUX4 also suppresses transcription of some target genes.

To investigate whether DUX4 operates solely as a transcriptional activator, we considered *t*-values derived from differential expression analysis of dataset GSE77100 by comparing gene expression driven by DUX4, tMALDUX4, tMALDUX4–VP16, tMALDUX4–ERD and DUX4c to that with control retrovirus (Fig. 5A). DUX4 and tMALDUX4–VP16 displayed a strong positive correlation in their respective transcriptional differential expression values from control (*r*=0.835, *P*<2.2×10^−16^), showing that *DUX4* is a transcriptional activator (Fig. 5B). tMALDUX4–VP16 and tMALDUX4–ERD had anti-correlated transcriptional perturbations from control, which is unsurprising because ERD and VP16 domains mediate inverse transcriptional responses (*r*= – 0.087, *P*<2.2×10^−16^). Importantly, DUX4 and tMALDUX4–ERD displayed no correlation in their transcriptional perturbations from control (*r*=0.007, *P*=0.15), suggesting that DUX4 also suppresses transcription of some target genes (Fig. 5B).

Transcriptional changes elicited by DUX4c were positively correlated with those caused by DUX4 (*r*=0.41, *P*<2.2×10^−16^), indicating overlap in their transcriptional influence (Fig. 5B). Interestingly, DUX4c-induced transcriptional changes were significantly positively correlated with those induced by both tMALDUX4–VP16 (*r*=0.55, *P*<2.2×10^−16^) and tMALDUX4–ERD (*r*=0.04, *P*=3.5×10^−12^), suggesting that although DUX4c increases transcription of some genes that are induced by DUX4, it might also repress a considerable proportion, acting in an antagonistic manner on the DUX4 phenotype. Finally, transcriptional changes caused by tMALDUX4 and DUX4c were highly correlated (*r*=0.817, *P*<2.2×10^−16^) (Fig. 5B), indicating that the unique 32-amino-acid C-terminus of DUX4c does not drastically alter its transcriptional profile.

### Concordance between DUX4-driven gene expression changes in mouse and humans

**Fig. 6.**
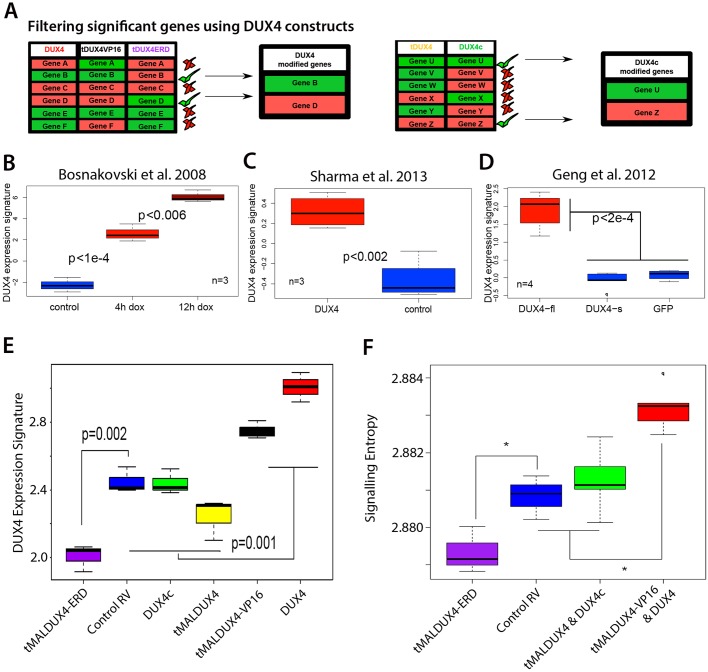
**DUX4 induces signatures of a stem-cell-like and less-differentiated state.** (A) Transcripts that were upregulated (red) by DUX4 and tMALDUX4VP16 (tDUX4VP16) but downregulated (green) by tMALDUX4ERD (tDUX4ERD) were considered as positively correlated (upregulated) with DUX4 activity. Conversely, transcripts that were downregulated (green) by DUX4 and tMALDUX4VP16 but upregulated (red) by tMALDUX4ERD were considered as negatively correlated (downregulated) with DUX4 activity. Transcripts upregulated (red) by tMALDUX4 and DUX4c were considered as positively correlated (upregulated) with DUX4c activity. Conversely, transcripts downregulated (green) by tMALDUX4 and DUX4c were considered as negatively correlated (downregulated) with DUX4c activity. (B–D) We constructed a single-sample DUX4 expression score from our study in mouse to examine overlap with DUX4 target genes identified by other studies. (B,C) Our mouse DUX4 expression score distinguishes murine C2C12 myoblasts expressing DUX4 from controls in two independent published microarray studies (Bosnakovski et al., 2008b; Sharma et al., 2013). (D) Our mouse DUX4 expression score also distinguishes DUX4-expressing human immortalised myoblasts from those expressing DUX4-s or eGFP control (Geng et al., 2012). (E) A human DUX4 signature derived from human myoblasts expressing DUX4 (Geng et al., 2012) distinguishes mouse myoblasts expressing tMALDUX4 and DUX4c both from those expressing DUX4 or tMALDUX4–VP16, and also from those expressing tMALDUX4–ERD. (F) Signalling entropy is elevated in the transcriptional profiles induced by DUX4 and tMALDUX4–VP16 but is reduced by tMALDUX4–ERD expression, supporting the hypothesis that DUX4 inhibits myogenic differentiation. Boxes represent the interquartile range (central 50% of data) with the median indicated by a line, and whiskers indicate the extremes of the distribution. *P*-values were calculated using Student's *t-*test. RV, retrovirus.

We also performed differential expression analyses using an empirical Bayes approach employing a *P*<0.05 significance threshold (Smyth, 2004) in comparing gene expression in the presence of DUX4, tMALDUX4, tMALDUX4–VP16, tMALDUX4–ERD and DUX4c independently to that under control retrovirus (Fig. 6A). A gene was considered upregulated by DUX4 if it was upregulated by both DUX4 and tMALDUX4–VP16 but downregulated by tMALDUX4–ERD, compared to control (Fig. 6A). A gene was considered downregulated by DUX4 if it was downregulated by both DUX4 and tMALDUX4–VP16 but upregulated by tMALDUX4–ERD, compared to control (Fig. 6A). Together, this generated a sample-specific biomarker for DUX4 activity by comparing expression of 291 DUX4-upregulated target genes to 344 DUX4-downregulated target genes in each sample (Table S1). DUX4-upregulated target genes should be at higher levels than downregulated target genes in samples expressing DUX4; thus, the difference between upregulated and downregulated target gene distribution is a biomarker for DUX4 expression.

This DUX4 biomarker shows significant concordance in the genes changed by DUX4 in our microarray using primary mouse satellite cells and those identified as changed in C2C12 myoblasts (Bosnakovski et al., 2008b; Sharma et al., 2013) (Fig. 6B,C). Importantly, this DUX4 biomarker also shows significant concordance with changes elicited by DUX4 in human cells (Geng et al., 2012), and can be used to distinguish human myoblasts expressing DUX4 from those expressing either DUX4-s or control (Fig. 6D).

Using the microarray analysis of human myoblasts that expressed DUX4 (Geng et al., 2012), we also determined the DUX4 transcription signature in humans comprising 123 upregulated and 253 downregulated genes (Table S2). This human DUX4 signature clearly separated our DUX4- and tMALDUX4–VP16-expressing mouse myoblasts from those expressing DUX4c and tMALDUX4 (Fig. 6E), and also those expressing tMALDUX4–ERD from DUX4c- and tMALDUX4-expressing myoblasts (Fig. 6E). Thus, genes controlled by DUX4 in mouse overlap with those regulated by DUX4 in humans.

### DUX4 increases transcriptomic measures of stem cells

Signalling entropy is a combined single-sample measure of intracellular signalling promiscuity and intercellular heterogeneity, derived from integration of gene expression data with a protein interaction network. Signalling entropy is a powerful measure of cell differentiation potential, valid across multiple lineages and in pathology, and we have shown previously that it outperforms other popular methodologies (Banerji et al., 2013, 2015b). The assumption is that stem cells have many options with respect to fate, and so the diversity of genes expressed is high, giving stem cells a high signalling entropy. In contrast, differentiated cells have a more limited and defined gene expression profile in order to perform their functions, so have low signalling entropy. Thus signalling entropy progressively drops during progress from stem cells to differentiated cells, so signalling entropy indicates the position of a cell population on this spectrum (Banerji et al., 2013).

Computing signalling entropy for each DUX4 construct revealed that gene expression profiles induced by DUX4 and tMALDUX4–VP16 displayed significantly higher signalling entropy than those induced by control (*P*<0.005) and DUX4c (*P*<0.0006), suggesting that DUX4 results in a transcriptomic profile that is more like that of a stem cell or of a less-differentiated cell (Fig. 6F). In contrast, tMALDUX4–ERD displayed a significantly lower signalling entropy than control (*P*<0.04), suggesting that repression of DUX4 target genes causes a more differentiated expression regime. tMALDUX4- and DUX4c-expressing cells had similar signalling entropy to that of control cells (Fig. 6F), suggesting that they do not significantly alter global transcriptomic measures of differentiation potential, despite their effects on key markers of differentiation at the protein level (Figs 3 and 4).

### DUX4 regulates genes associated with apoptosis and reduced cell proliferation

DUX4 principally activates transcription of target genes, whereas DUX4c and tMALDUX4 activate some of these DUX4 target genes but repress others. We compared pathways that are regulated by DUX4 and DUX4c using sequential gene-set filtering and information from the four DUX4 construct and DUX4c microarrays (Fig. 6A). In addition to the target gene set that acts as a DUX4 biomarker, we also generated two DUX4c target gene sets – one in which genes were considered to be upregulated by DUX4c if they were upregulated by both tMALDUX4 and DUX4c, and one in which genes were considered to be downregulated by DUX4c if they were downregulated by both tMALDUX4 and DUX4c (Fig. 6A). Gene set enrichment analysis was used to evaluate whether genes that were commonly and differentially regulated by DUX4 and DUX4c (Table S3) were significantly associated with particular functional classes. After correcting for multiple testing, there was no enrichment for gene sets that were downregulated by both DUX4 and DUX4c or that were upregulated by DUX4 but not DUX4c (Tables S4 and S5).

**Fig. 7.**
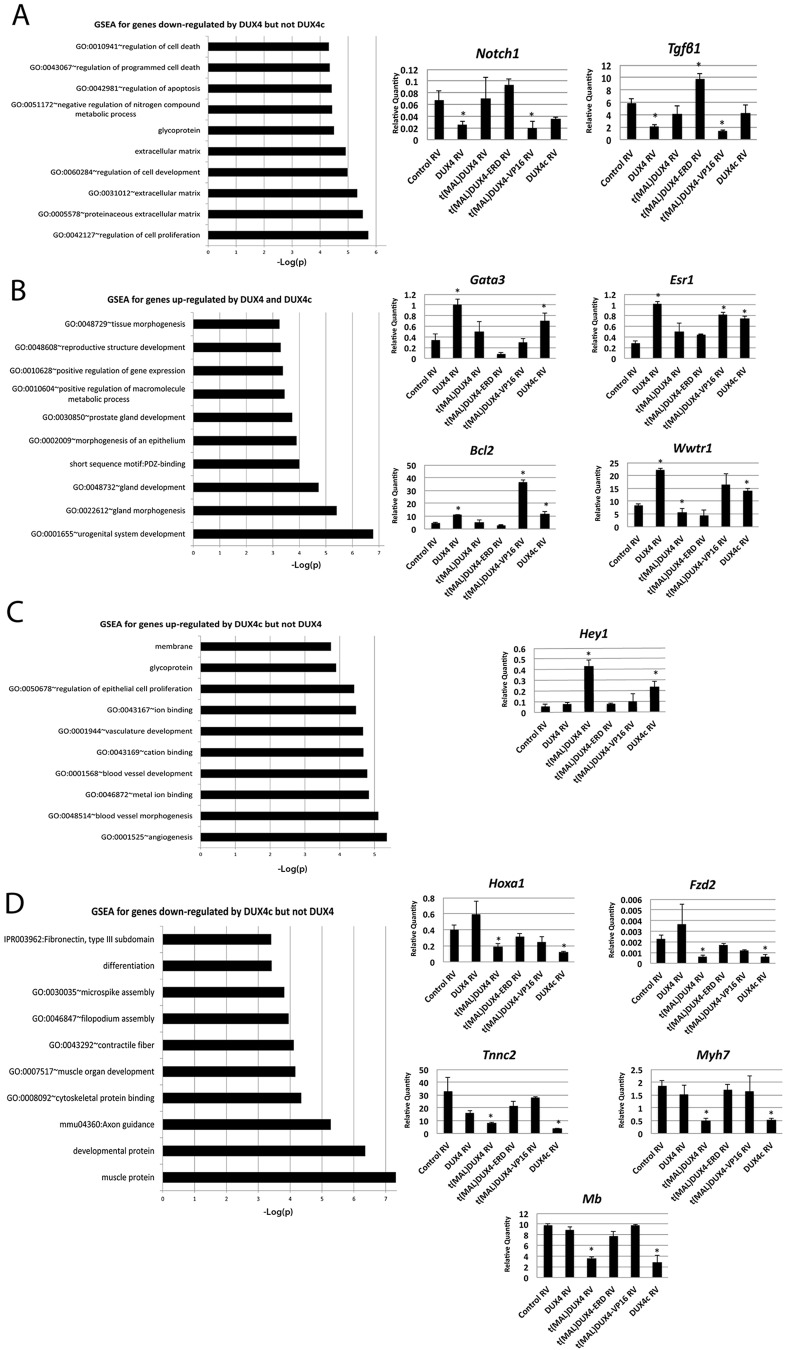
**GSEA reveals pathways regulated by DUX4 and DUX4c.** (A) GSEA for genes downregulated by DUX4 but not DUX4c, with RT-qPCR validation for genes encoding Notch 1 and TGFβ1, relative to *Tbp* expression. (B) GSEA for genes upregulated by both DUX4 and DUX4c with RT-qPCR validation for *Gata3*, *Esr1*, *Bcl2* and *Wwtr1*, relative to *Tbp* expression. (C) GSEA for genes upregulated by DUX4c but not by DUX4, with RT-qPCR validation for *Hey1*, relative to *Tbp* expression. (D) GSEA for genes downregulated by DUX4c but not by DUX4, with RT-qPCR validation for *Hoxa1*, *Fzd2*, *Tnnc1*, *Myh7* and *Mb* (myoglobin)*.* Data are mean±s.e.m. using myoblasts from four mice, where an asterisk denotes significant difference from control using a paired Student's *t*-test (*P*<0.05). RV, retrovirus.

Crucially, genes downregulated by DUX4 but not DUX4c were significantly enriched for those regulating cell proliferation and apoptosis, for example those encoding TGFβ1 and Notch1 (Fig. 7A; Table S6). Genes upregulated by both DUX4 and DUX4c were significantly enriched for urogenital development and gland development, for example *Gata3*, *Esr1*, *Bcl2* and *Wwtr1* (Fig. 7B; Table S7). Genes that were upregulated by DUX4c but not DUX4 were strongly associated with angiogenesis and blood vessel morphogenesis, for example *Hey1* (Fig. 7C; Table S8). Conversely, genes downregulated by DUX4c but not DUX4 were associated with developmental processes and muscle development, for example *Hoxa1*, *Fzd2*,* Tnnc2*, *Myh7* and myoglobin (*Mb*) (Fig. 7D; Table S9).

## DISCUSSION

*DUX4* plays a key role in FSHD1 and FSHD2 pathology because of its de-repression in skeletal muscles (Tawil et al., 2014). Epigenetic regulation of the D4Z4 repeat in transgenic D4Z4-2.5 mice is generally similar to that in man, with variable low levels of DUX4 in skeletal muscle, but the transgenic model has no overt muscle pathology (Krom et al., 2013). Here, we show that *DUX4* expression increases during muscle regeneration, being expressed by myoblasts, although overall, DUX4 levels remained low. Our observations are consistent with those made in primary FSHD myoblasts, where both DUX4 and its transcriptional activity can be detected in proliferating and differentiating human myoblasts (Dixit et al., 2007; Jones et al., 2012; Kowaljow et al., 2007; Rickard et al., 2015; Snider et al., 2010). Mice have an impressive regeneration capacity, and so low DUX4 levels or expression restricted to a few myoblasts might explain the lack of an overt muscle phenotype. The *DUX4* locus is predisposed to being expressed and is activated by, amongst other things, myogenic transcriptional regulators. Recently, two myogenic enhancers have been identified (Himeda et al., 2014), one of which, the *DUX4* myogenic enhancer 1 (*DME1*), is included in the D4Z4-2.5 transgene.

*DUX4* splice variants emanate from the D4Z4 repeat array (Snider et al., 2009), and inappropriate temporal expression or increased proportions of the transcript encoding DUX4-fl are probably pathogenic in FSHD muscle. DUX4-fl and splice variants inhibit myoblast differentiation (Bosnakovski et al., 2008b; Snider et al., 2009), and DUX4-fl is also apoptotic (Bosnakovski et al., 2008b; Mitsuhashi et al., 2013; Wallace et al., 2010). DUX4-fl contains double homeobox DNA-binding domains and an evolutionarily conserved peptide sequence at the C-terminus (Clapp et al., 2007) that acts as a strong transcriptional activation domain (Kawamura-Saito et al., 2006). To better understand the mode of action of DUX4 and DUX4c on myogenesis, we used our panel of four DUX4 constructs, including constitutively active, dominant-negative and truncated versions of DUX4.

Pax7 is expressed in activated satellite cells, but levels decrease during differentiation, with Pax7 and myogenin expression being mutually exclusive (Zammit et al., 2004). DUX4 and tMALDUX4–VP16 resulted in maintenance of *Pax7* expression, as did DUX4c, whereas transcriptional repression of target genes by the tMALDUX4–ERD construct did not alter *Pax7* levels. DUX4, tMALDUX4 and DUX4c also reduced *Myod* expression. Because tMALDUX4–VP16 but not tMALDUX4–ERD reduced *Myod* levels, it is likely that DUX4 activates genes involved in *Myod* repression rather than by directly repressing *Myod* transcription itself, providing insight into MYOD-dependent pathway suppression in FSHD (Celegato et al., 2006; Winokur et al., 2003b).

Interestingly, the C-terminal peptide of DUX4 inhibits myogenin expression in the absence of the DNA-binding homeodomains (Snider et al., 2009). tMALDUX4–VP16 does not contain this C-terminal peptide and did not alter myogenin gene expression, showing that DUX4 is not solely acting by transcriptionally activating target genes, consistent with observations that tMALDUX4–ERD also suppresses myogenin. *Myf5* mRNA is upregulated by DUX4 in immortalised myoblasts and satellite cells (Banerji et al., 2015a; Bosnakovski et al., 2008b) and this could represent a compensatory mechanism. However, DUX4 inhibits both *Myod* and myogenin gene expression in mouse satellite cells to produce a differentiation defect that cannot be overcome by upregulation of *Myf5*.

All DUX4 constructs and DUX4c inhibited myoblast fusion into multinucleated myotubes, but DUX4c and tMALDUX4 had relatively mild effects. Myoblasts were re-plated at high-density before assessing fusion, to mitigate the effects on proliferation. However, tMALDUX4–ERD did not affect proliferation yet still blocked fusion, indicating that transcriptional activation of DUX4 target genes inhibits proliferation, but both activation and suppression of target genes can suppress differentiation.

Thus, DUX4 expression results in maintenance of a stem-cell-like and less-differentiated state, with concomitant suppression of proliferation and inhibition of differentiation. This striking differentiation defect might explain the lack of muscle phenotype in our D4Z4-2.5 mice because rare DUX4-expressing myoblasts might be inhibited from fusing into myofibres.

To better understand DUX4, we further analysed our microarray of satellite-cell-derived myoblasts expressing DUX4, tMALDUX4–VP16, tMALDUX4–ERD, tMALDUX4 or DUX4c constructs (Banerji et al., 2015a). Pairwise comparison of the transcriptional changes caused by each construct compared to control allowed us to determine the predominant mode of action of DUX4. Transcriptional changes elicited by DUX4 or tMALDUX4–VP16 were strongly positively correlated, indicating that DUX4 activates many transcriptional targets. Interestingly, although DUX4 and tMALDUX4–VP16 had very similar transcriptome signatures, they were not identical, indicating that DUX4 is not operating solely as a transcriptional activator. Indeed, although the expression profile of tMALDUX4–VP16 target genes was anti-correlated to that of tMALDUX4–ERD, DUX4 was not, indicating that DUX4 also suppresses some transcriptional target genes. The target gene sets of tMALDUX4 and DUX4c were positively correlated, but were also positively correlated with DUX4, indicating that they have many target genes in common. This again suggests additional mechanisms by which DUX4 alters transcriptional regulation that are distinct from the activity of its C-terminal transactivation domain.

Signalling entropy is a strong correlate of differentiation potential in healthy tissue (Banerji et al., 2013) and is a powerful prognostic factor in cancerous tissue, where it is associated with anaplasia (Banerji et al., 2015b). tMALDUX4 or DUX4c induced similar signalling entropies to control, whereas tMALDUX4–ERD decreased signalling entropy, indicating induction of differentiation. In contrast, signalling entropy was raised by DUX4 or tMALDUX4–VP16, implying that DUX4 activates transcriptional target genes that are expressed in stem cell populations, consistent with retention of Pax7 expression in satellite cells.

Although there are Dux-like genes in mouse, there is debate about how useful mouse studies are for identifying genes regulated by DUX4. However, there has only been limited assessment of the concordance in mouse and man between DUX4-mediated transcriptional changes. There was a 27% overlap of transcripts that are differentially expressed by DUX4 in mouse C2C12 myoblasts compared to human RD rhabdomyosarcoma cells expressing DUX4, despite effects associated with comparing mouse myoblasts with human cancer cells (Sharma et al., 2013). We have also demonstrated previously a 23% overlap in DUX4 targets between mouse and man using the transgenic D4Z4-2.5 mouse model (Krom et al., 2013). However, the significance of this overlap in DUX4-perturbed genes was not statistically assessed in these studies.

Objectively assessing mouse as a FSHD model is requisite because many mouse models have been developed for FSHD (Lek et al., 2015). Reliable transcriptomic profiling of DUX4 overexpression requires matched cell types between mouse and man, and statistical assessment of target overlap. We developed a DUX4 signature of genes using our microarray in primary mouse satellite cells. Our mouse DUX4 signature could distinguish mouse C2 myoblasts expressing DUX4 from control cells, described in two independent studies (Bosnakovski et al., 2008b; Sharma et al., 2013). Importantly, this overlap extended to a human microarray study (Geng et al., 2012), where genes identified as being perturbed by DUX4 in our murine myoblasts could also be used to distinguish human myoblasts overexpressing DUX4 from those expressing DUX4-s or eGFP controls. We also derived a human DUX4 signature, which separated our DUX4- and tMALDUX4–VP16-expressing mouse myoblasts from those expressing DUX4c and tMALDUX4, and also tMALDUX4–ERD-expressing cells from those expressing DUX4c and tMALDUX4*.* Thus, there is a statistically significant overlap in DUX4 transcriptional dysregulation across mouse and man. Furthermore, DUX4 in mouse primary myoblasts perturbs expression of genes that are modified in multiple human FSHD muscle biopsies (Banerji et al., 2015a).

Using transcriptome data from mouse satellite cells expressing DUX4 or tMALDUX4–VP16, we isolated genes that are likely to be transcriptionally activated by DUX4. Identifying those genes that exhibited inverse expression patterns in satellite cells expressing tMALDUX4–ERD increases the confidence that they are pathways regulated by DUX4. However, DUX4c or DUX4 splice variants also perturb myoblast function (Ansseau et al., 2009; Bosnakovski et al., 2008b; Snider et al., 2009). Using the four DUX4 constructs and DUX4c, we filtered gene expression profiles to provide sets of genes that are perturbed by DUX4 and/or DUX4c. As expected, those genes regulated by DUX4 but not DUX4c were enriched for genes involved in apoptosis and proliferation, consistent with observations that DUX4, but not DUX4c, is pro-apoptotic in myoblasts. DUX4c-enriched genes were involved in vascular development, which is relevant given an association with Coat's like retinopathy and FSHD (Fitzsimons, 2011). DUX4c-perturbed genes are also involved in muscle development, supporting an active role for DUX4c in FSHD muscle pathology (Ansseau et al., 2009). Both DUX4 and DUX4c regulate genes expressed during urogenital and gland morphogenesis, supporting DUX4 expression in testes and indicating that overlapping DUX4 and DUX4c transcriptional targets could guide development of urogenital organs (Snider et al., 2010). Finally, genes downregulated by DUX4c but not DUX4 were associated with muscle development and axonal guidance. Both DUX4 and DUX4c inhibit myoblast fusion, whereas DUX4 overexpression in embryonic stem cells promotes differentiation towards the neuronal lineage (Dandapat et al., 2014), indicating that DUX4c is associated with neuronal and myogenic development in a manner that is independent of DUX4. These transcriptome signatures add to our understanding of how DUX4 and DUX4c induce pathology in FSHD. Examining multiple DUX4 constructs also allows for the identification of target genes that could be overlooked when examining DUX4 alone due to its effects on proliferation and apoptosis.

Overall, our study suggests that induction of a more a stem-cell-like and less-differentiated state in myoblasts expressing DUX4 inhibits proliferation and myogenesis. Identification of pathways perturbed by DUX4 contributes to the challenge to identify viable therapeutic targets to alleviate the consequences of mis-expression of DUX4 in FSHD.

## MATERIALS AND METHODS

### Muscle injury

Procedures were carried out under the Animals (Scientific Procedures) Act 1986, as approved by King's College London Ethical Review Process committee or approved by the local animal experimental committee of Leiden University Medical Center and by the Commission Biotechnology in Animals of the Dutch Ministry of Agriculture. Four-month-old hemizygous D4Z4-2.5 and D4Z4-12.5 mice were used (Krom et al., 2013). Muscle injury was induced by intra-muscular injection of 10 μM cardiotoxin in 50 μl PBS into the gastrocnemius of anaesthetised mice. Contra-lateral muscles were injected with 50 μl saline. Muscles were isolated at days 3, 4, 5, 6 and 10 post-cardiotoxin, snap-frozen in 2-methylbutane (Sigma-Aldrich, Dorset, UK) cooled in liquid nitrogen, cryosectioned and stained with haematoxylin and eosin (H&E). D4Z4-2.5 (stock #027991) and D4Z4-12.5 (stock #028012) transgenic mice are available from the Jackson Laboratory.

### qPCR

RNA was isolated using miRNeasy kit (Qiagen, Manchester, UK) including DNase digestion. Tissues were homogenised in 700 μl qiazol and resuspended in 700 μl qiazol. RNA quality and concentration were checked by using a LabChip Bioanalyzer (Agilent) and Nanodrop (ND-1000 spectrophotometer, Thermo Fisher Scientific). cDNA synthesis was performed with 3 μg RNA using Revert Aid H minus first strand cDNA synthesis kit (Thermo Fisher Scientific) and oligo dT primers (Thermo Fisher Scientific). Control comprised no RevertAid H minus M-MulV and Ribolock RNase inhibitor samples. SYBR-Green-based real-time PCR (96°C for 6 min, 40 cycles at 95°C for 10 s, 60°C for 30 s, 95°C for 10 s, followed by melt curve analysis) on a CFX96 system (BioRad, Hertfordshire, UK). Primers were designed using Primer3 software: DUX4-Fw, 5′-CCCAGGTACCAGCAGACC-3′, Rev, 5′-CCCAGGTACCAGCAGACC-3′; *Myog*-Fw, 5′-CCTTGCTCAGCTCCCTCA-3′, Rev, 5′-TGGGAGTTGCATTCACTGG-3′; MyoD-Fw, 5′-TACAGTGGCGACTCAGATGC-3′, Rev, 5′-TAGTAGGCGGTGTCGTAGCC-3′; α7-integrin (*Itga7*)-Fw, 5′-CCTGGAAGTGATCGTCCGAG-3′, Rev, 5′-CCATGGGGTCCAAGTACACC-3′; *Ptprc* (*Cd45*)-Fw, 5′-CCTGCAGAACCCAAAGACCT-3′, Rev, 5′-CCTGTCTGCTGGGATCCATC-3′; *Duxbl-*Fw, 5′-GCATCTCTGAGTCTCAAATTATGACTTG-3′, Rev, 5′-GCGTTCTGCTCCTTCTAGCTTCT-3′; *Tbp*-Fw, 5′-CTCAGTTACAGGTGGCAGCA-3, Rev, 5′-CAGCACAGAGCAAGCAACTC; *RPL13*a-Fw, 5′-GCTGCTCTCAAGGTTGTTC-3′, Rev, 5′-TTCTCCTCCAGAGTGGCTGT-3′; *Notch1*-Fw, 5′-TCAATGTTCGAGGACCAGATG-3′, Rev, 5′-TCACTGTTGCCTGTCTCAAG-3′; *Tgfb1*-Fw, 5′-CCCTATATTTGGAGCCTGGA-3′, Rev, 5′-CTTGCGACCCACGTAGTAGA-3′; *Hey1*-Fw, 5′-TACCCAGTGCCTTTGAGAAG-3′, Rev, 5′-AACCCCAAACTCCGATAGTC-3′; *Tnnc2*-Fw, 5′-CGAGGATGGCAGCGGTACTA-3′, Rev, 5′-CCTTCGCATCCTCTTTCATCTG-3′; *Myh7*-Fw, 5′-CCAAGAAGGCTATCACAGATGC-3′, Rev, 5′-TTCCTGTCTTCCTCTGTCTGGT-3′; *Mb*-Fw, 5′-GGCAGCTGGTGCTGAATGT-3′, Rev, 5′-TAAACAGACCGATGAGGACTTCCT-3′; *Fzd2*-Fw, 5′-TCGCCTACAACCAGACCATC-3′, Rev, 5′-CATTGGAAGCCGAACTTGT-3′; *Hoxa1*-Fw, 5′-CTTCTCCAGCGCAGACCTT-3′, Rev, 5′-CTGTGAGCTGCTTGGTGGT-3′; *Gata3*-Fw, 5′-TTTACCCTCCGGCTTCATCCTCCT-3′, Rev, 5′-TGCACCTGATACTTGAGGCACTCT-3′; *Esr1*-Fw, 5′-GCACAGGATGCTAGCCTTGTCTC-3′, Rev, 5′-CCAGCTTGCAGGTTCATTGTG-3′; *Bcl2*-Fw, 5′-TGAGTACCTGAACCGGCATCT-3′, Rev, 5′-GCATCCCAGCCTCCGTTAT-3′; *Wwtr1*-Fw, 5′-GCCACTGGCCAGAGATACTT-3′, Rev, 5′-GACGGGTGGAGGTTCACAT-3′.

### FACS

Gastrocnemius muscles were isolated four days after cardiotoxin injection. Two control and two cardiotoxin-injected muscles were frozen to assess DUX4 levels. For FACs, cardiotoxin-injected muscles were minced and digested in 1.2 units/ml dispase II, 2 mg/ml collagenase type IV (Worthington) and 2 mM CaCl_2_ in PBS for 45 min at 37°C. Enzymes were neutralised with HAM'S/F10 with 15% horse serum and passed through a 70-µm then 40-µm nylon cell strainer (BD Falcon, Oxfordshire, UK). Samples were centrifuged at 300 ***g*** for 5 min and the pellet was re-suspended in haemolytic buffer (155 mM NH_4_Cl, 10 mM KHCO_2_ and 0.1 mM EDTA) for 5 min at room temperature before centrifugation at 300 ***g*** for 5 min. The pellet was resuspended in 6 ml PBS with 0.5% BSA. Cells were immunostained with anti-CD45 Alexa-Fluor-700-conjugated antibody (eBioscience, Hertfordshire, UK), anti-CD31 PE-cyanine-7-conjugated antibody (eBioscience), anti-SCA1 efluor605NC-conjugated antibody (eBioscience), anti-α7-integrin FITC-conjugated antibody (R&D Systems, Oxfordshire, UK) and diluted in 100 µl PBS with 0.5% BSA per 10^6^ cells for 45 min on ice and washed twice in PBS with 0.5% BSA with centrifugation between washes. Cells were resuspended in 1.5 ml PBS with 0.5% BSA, filtered and stored on ice. 5.3×10^6^ CD45^+^cells and 1.5×10^6^ CD31^−^ CD45^−^ SCA1^−^ α7 integrin^+^ cells were analysed by FACS with an AriaIII FACS instrument and centrifuged at 400 ***g*** for 15 min at 4°C.

### Myofibres and satellite cells

C57BL/10 male mice (6–8 weeks) were killed, and EDL muscles were dissected, and myofibres liberated by enzymatic digestion (Moyle and Zammit, 2014). Myofibres with their associated satellite cells were transferred to 5% BSA (Sigma-Aldrich) coated dishes and cultured at 37°C with 5% CO_2_ in DMEM Glutamax (Thermo Fisher Scientific) with 10% horse serum (v/v) (Gibco), 0.5% chick embryo extract (CEE) (v/v) and 1% penicillin-streptomycin (v/v) (Sigma-Aldrich).

### Satellite-cell-derived myoblast preparation

Myofibres were plated at ∼100 fibres/well in 6-well plates coated with 1 mg/ml Matrigel (Collaborative Research). Muscle fibres were cultured in medium comprising DMEMGlutamax (Thermo Fisher Scientific) with 30% foetal bovine serum (FBS) (v/v), 10% horse serum, 1% CEE, 10 ng/ml basic fibroblast growth factor (Peprotech, London, UK), 1% penicillin-streptomycin at 37°C with 5% CO_2_ for 72 h. Myofibres were removed and myoblasts expanded for 48 h. To induce differentiation, myoblasts were cultured in DMEMGlutamax with 2% HS (v/v) and 1% penicillin-streptomycin (v/v).

### DUX4 constructs

DUX4, DUX4c, tMALDUX4, tMALDUX4–VP16 and tMALDUX4–ERD were encoded in pMSCV-IRES-eGFP (Banerji et al., 2015a). An IRES preceding eGFP allows independent translation to identify transduced cells. All constructs were sequenced.

Retrovirus was produced by co-transfecting pMSCV-IRES-eGFP DUX4 cDNA's and an ecotropic helper plasmid into HEK293T using Lipofectamine (Thermo Fisher Scientific). pMSCV-IRES-eGFP was control.

### Retroviral transduction

Myofibres maintained in DMEMGlutamax with 10% horse serum (v/v), 0.5% CEE (v/v) and 1% penicillin-streptomycin (v/v) in 5%-BSA-coated 6-well plates at 37°C, 5% CO_2_ for 24 h before transduction. Non-adherent myofibres were maintained at 37°C 5% CO_2_ for 24 or 48 h post-transduction. Satellite-cell-derived myoblasts were re-plated at 1.5×10^5^ cells per Matrigel-coated well of a 6-well plate (Collaborative Research). Cells were maintained in high-serum medium for 24 h before medium replacement and transduction 1 h later. Cells were incubated at 37°C 5% CO_2_ with retrovirus for 4 h before medium replacement to DMEMGlutamax with 30% FBS (v/v), 10% horse serum, 1% CEE, 1% penicillin-streptomycin. After 24 h, cells were re-plated at 5×10^3^ (for proliferation) or 2.5×10^4^ (for differentiation) cells/well in Matrigel-coated chamber slides.

### Immunostaining

Myofibres and myoblasts were fixed in 4% paraformaldehyde, permeabilised in 0.5% Triton-X (Sigma-Aldrich) for 10 min, washed with PBS then blocked for 30 min in 5% (v/v) swine and goat serum (DakoCytomation Glostrup, Denmark), incubated in primary antibodies overnight at 4°C [mouse anti-DUX4 antibody [9A12 mAb, a kind gift from Alexandra Belayew (University of Mons, Mons, Belgium), 1:2000], mouse anti-Pax7 antibody [AB528428, Developmental Studies Hybridoma Bank (DSHB); 1:20–1:100], mouse anti-MyoD antibody clone 5.8A (M3512, DakoCytomation; 1:50), mouse anti-myogenin antibody (F5D, DSHB; 1:15–1:50), mouse anti-MyHC antibody (MF20, DSHB; 1:400), rabbit anti-GFP antibody (A-11122, Thermo Fisher Scientific; 1:1000), rabbit anti-phospho-histone-H1 (06-597, Millipore, 1:300) and anti-phospho-histone-H3 antibodies (06-570, Millipore; 1:100). After washing, incubation for 1 h at room temperature with Alexa-Fluor-conjugated secondary antibodies (Thermo Fisher Scientific) (Moyle and Zammit, 2014).

Images were acquired on a Zeiss Axiovert 200 M microscope using a Zeiss AxioCamHRm and AxioVision version 4.4 (Zeiss) or a Zeiss Axioplan 2 with a Hamamatsu ORCA-ER camera with Openlab 3.1.7.

### EdU incorporation

Myoblasts were plated at 5×10^3^ in 8-well Matrigel-coated chamber slides, maintained in high-serum medium for 24 h before transduction, and 24 h later, pulsed with EdU for 2 h (Thermo Fisher Scientific) and immunostained for eGFP before EdU detection with Alexa-Fluor-594 (Thermo Fisher Scientific).

### Reporter gene assay

C2C12 myoblasts were co-transfected using Lipofectamine LTX (Thermofisher) with DUX4c or DUX4 constructs or control GFP and DUX4-responsive promoters driving luciferase (pZSCAN4-luc, pKHDC1L-luc, *RFPL4b-luc*), together with a pRSV-β-galactosidase construct to normalise transfections. Myoblasts were harvested 24 h later, and assayed using the Dual-light Reporter system (Thermofisher) in three transfections measured in triplicate on a Glomax-Multi+ plate reader (Promega).

### Apoptosis assay

Transduced myoblasts were plated (5×10^3^/well) into 96-well plates for fluorescence assays (Greiner Bio-One) in three technical replicates to investigate apoptosis using the Caspase-Glo 3/7 Assay (Promega) on a Glomax-Multi+ microplate reader (Promega). Luminescence activity from the Caspase-Glo assay from each well was normalised to GFP measured using the Glomax-Multi+ reader.

### Statistical analysis

Myofibre and satellite-cell-derived myoblasts were obtained from at least three mice. Data from immortalised myoblast lines was from at least three experiments. Data are mean±s.e.m. with significance assessed by Student's *t*-test, unless otherwise stated.

### DUX4 microarray analysis

GSE77100 microarray is available from http://www.ncbi.nlm.nih.gov/geo/query/acc.cgi?acc=GSE77100 (Banerji et al., 2015a). Acquisition and normalisation of these microarray data has been previously described (Banerji et al., 2015a). Briefly, expanded satellite-cell-derived myoblasts from three male 8-week-old C57BL/10 mice were transduced with retroviruses encoding DUX4, DUX4c, tMALDUX4, tMALDUX4–VP16, tMALDUX4–ERD or control pMSCV-IRES-eGFP with 4 mg/ml polybrene for 20 h. RNA was extracted using Qiagen RNeasy Kit and quantified. Gene expression analysis was performed using GeneChip Mouse Gene 1.0ST Array and GCS3000 microarray system (Affymetrix) by the King's Genomic Centre.

Differential expression analysis was performed using an empirical Bayes approach (Smyth, 2004) to identify transcripts perturbed by each DUX4 construct, *t*-statistics for transcripts were correlated between constructs to ascertain similarities in expression landscapes. *t*-values described in reference to differential expression are the test statistics of a standard statistical assessment of differential expression using the Linear Models for Microarrays (limma) package in R (Smyth, 2004). Transcripts were filtered using all constructs to obtain two lists representing genes whose expression was modified by either DUX4 or DUX4c. *P*<0.05 was used to identify genes which were differentially expressed by each DUX4 construct compared to control. Expression of genes was then attributed as DUX4 upregulated if they were upregulated by both DUX4 and tMALDUX4–VP16 and downregulated by tMALDUX4–ERD. Expression of genes was attributed as DUX4 downregulated if they were downregulated by both DUX4 and tMALDUX4–VP16 and upregulated by tMALDUX4–ERD. Similarly, a gene was considered to be up- or downregulated by DUX4c if it was up- or downregulated by both DUX4c and tMALDUX4.

GSEA was performed using a Fisher's Exact test, using the DAVID functional annotation tool (Huang et al., 2009a,b). Gene sets which displayed Benjamini–Hochberg adjusted *P*<0.05 were considered enriched.

### Signalling entropy

Signalling entropy was computed using a mass action principle approximation (Banerji et al., 2013). Each sample was integrated with a protein interaction network (PIN) to create a sample specific stochastic matrix, *P=(p_ij_)*. The PIN was constructed from previous work (Banerji et al., 2015b) through orthology relations. The *i*^th^ row of *P* defines a probability distribution describing rates of reaction of protein *i* with each of its neighbours. Distributions were constructed appealing to a mass action principle, namely that rate of a reaction is proportional to the product of the active masses. Assuming log normalised gene expression is a proxy for protein concentration, we compute:

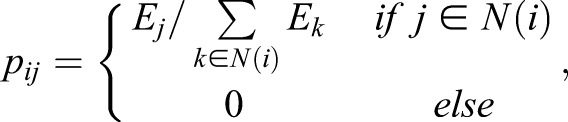
where *E_j_* is log-normalised expression of gene *j* in the given sample and *N*(*i*)** denotes the set of direct interaction partners of gene *i* in the PIN. From this definition, ∑*_j_*_ɛ*N*(*i*)_*P_ij_*=1 for all *j* – i.e. *P* is row stochastic and *i*^th^ row corresponds to weighted interaction distribution of protein *i* in sample. Not all proteins in the PIN have a corresponding microarray probe, consequentially the PIN is the maximally connected component after removal of missing proteins.

For each protein *i*, we define the local entropy of its interaction distribution, *S*_i_, quantifying promiscuity in its signalling within the sample:

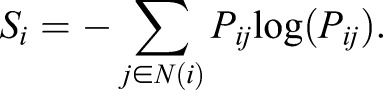
Signalling entropy is a global measure of signalling promiscuity and is computed from the stochastic matrix *p*_ij_ as the entropy rate (SR) of the stochastic process described by *p*_ij_:

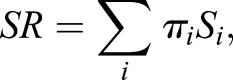
where *π_i_* denotes the stationary distribution of the stochastic matrix, satisfying:

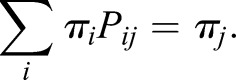
*π_i_* is the non-degenerate eigenvector of *P* corresponding to eigenvalue 1. By Perron–Frobenius existence of *π_i_* requires that matrix *P* be irreducible; as the PIN considered is connected and non-bipartite, this is guaranteed. R-scripts for signalling entropy can be found at www.sourceforge.net/projects/signalentropy.
